# m6A Regulators in Human Adipose Tissue - Depot-Specificity and Correlation With Obesity

**DOI:** 10.3389/fendo.2021.778875

**Published:** 2021-12-07

**Authors:** Torunn Rønningen, Mai Britt Dahl, Tone Gretland Valderhaug, Akin Cayir, Maria Keller, Anke Tönjes, Matthias Blüher, Yvonne Böttcher

**Affiliations:** ^1^ Clinical Molecular Biology (EpiGen), Division of Medicine, Akershus Universitetssykehus, Lørenskog, Norway; ^2^ Department of Clinical Molecular Biology (EpiGen), Institute of Clinical Medicine, University of Oslo, Oslo, Norway; ^3^ Department of Endocrinology, Akershus Universitetssykehus, Lørenskog, Norway; ^4^ Vocational Health College, Canakkale Onsekiz Mart University, Canakkale, Turkey; ^5^ Department of Medicine, University of Leipzig, Leipzig, Germany; ^6^ Helmholtz Institute for Metabolic, Obesity and Vascular Research (HI-MAG) of the Helmholtz Zentrum München at the University of Leipzig and University Hospital, Leipzig, Germany

**Keywords:** RNA methylome, m6A methylation, epitranscriptome, obesity, transcription, fat distribution

## Abstract

**Background:**

N^6^-methyladenosine (m6A) is one of the most abundant post-transcriptional modifications on mRNA influencing mRNA metabolism. There is emerging evidence for its implication in metabolic disease. No comprehensive analyses on gene expression of m6A regulators in human adipose tissue, especially in paired adipose tissue depots, and its correlation with clinical variables were reported so far. We hypothesized that inter-depot specific gene expression of m6A regulators may differentially correlate with clinical variables related to obesity and fat distribution.

**Methods:**

We extracted intra-individually paired gene expression data (omental visceral adipose tissue (OVAT) *N*=48; subcutaneous adipose tissue (SAT) *N*=56) of m6A regulators from an existing microarray dataset. We also measured gene expression in another sample set of paired OVAT and SAT (*N*=46) using RT-qPCR. Finally, we extracted existing gene expression data from peripheral mononuclear blood cells (PBMCs) and single nucleotide polymorphisms (SNPs) in *METTL3* and *YTHDF3* from genome wide data from the Sorbs population (*N*=1049). The data were analysed for differential gene expression between OVAT and SAT; and for association with obesity and clinical variables. We further tested for association of SNP markers with gene expression and clinical traits.

**Results:**

In adipose tissue we observed that several m6A regulators (*WTAP*, *VIRMA*, *YTHDC1* and *ALKBH5*) correlate with obesity and clinical variables. Moreover, we found adipose tissue depot specific gene expression for *METTL3*, *WTAP*, *VIRMA*, *FTO* and *YTHDC1.* In PBMCs, we identified *ALKBH5* and *YTHDF3* correlated with obesity. Genetic markers in *METTL3* associate with BMI whilst SNPs in *YTHDF3* are associated with its gene expression.

**Conclusions:**

Our data show that expression of m6A regulators correlates with obesity, is adipose tissue depot-specific and related to clinical traits. Genetic variation in m6A regulators adds an additional layer of variability to the functional consequences.

## Introduction

Obesity is a major health burden predisposing to a variety of serious co-morbidities including metabolic disorders (1). Increased fat storage in omental visceral adipose tissue (OVAT) strongly correlates with a higher risk of metabolic sequelae [such as insulin resistance, type 2 diabetes (T2D)], whilst accumulation of subcutaneous adipose tissue (SAT) is less correlated to metabolic alterations (2–4). Due to its multifactorial, complex nature, obesity is subject to regulation by genetic and non-genetic factors (5). In addition to dynamic epigenetic signatures regulating gene expression and function, epitranscriptomic regulation represent an additional layer of variability adding to the complexity of gene regulation and function (6). Among multiple mRNA modifications N6-methyladenosine (m6A) is the most abundant (7, 8) and is a reversible, epitranscriptomic mark, playing an essential role in RNA processing, particularly in regulating RNA stability and decay, RNA export, translation and splicing (9). Similar to DNA methylation and other dynamic epigenetic modifications, m6A is regulated by a number of regulators entitled as “writers”, “readers” and “erasers” (10). The abundance of m6A in mRNA is determined by the activity of the “writer” and “eraser” proteins. A multi-protein “writer” complex consisting of the two methyltransferases methyltransferase-like 3 and 14 (METTL3 and METTL14), along with several other co-regulators such as Wilms tumor 1-associated protein (WTAP), and vir like m6A methyltransferase associated (VIRMA) installs m6A predominantly at stop codons, 3´and 5´UTR regions (11). Further, m6A is dynamically removed by specific demethylases or “erasers” including fat mass and obesity associated (FTO) and AlkB homologue 5 (ALKBH5) (12, 13). Multiple “reader” proteins including YTH family proteins and insulin like growth factor 2 binding proteins (IGF2BPs) are responsible for translating m6A deposition into function (12). Readers such as YTHDF2 and YTHDF3 increase transcript decay, while in contrast IGFBPs (IGF2BP1, 2 and 3) function by stabilizing mRNA. Furthermore, YTHDF1 and YTHDF3 modify translation efficiency, while YTHDC1 influence alternative splicing and nuclear export of mRNA (14). The functional consequence of m6A is therefore highly dependent on the associated reader protein. Several studies indicated that m6A regulators are important players in metabolic diseases (15–18). Interestingly, genetic variants within the *FTO* gene are strongly associated with obesity (19) and robustly replicated in many studies (20). FTO-dependent m6A demethylation is functionally linked to adipogenesis in 3T3-L1 mouse adipocytes by overlooking alternative splicing (21). Zhao et al. (2014) further reported that m6A levels negatively correlate to *FTO* expression in adipogenesis and *FTO* siRNA knock down inhibits 3T3-L1 adipocyte differentiation. Recently, it was suggested that m6A modifications may be involved in adipose tissue expansion (22–24). So far, to the best of our knowledge, no attempts have been undertaken to investigate the role of m6A regulators in intra-individually paired samples of human visceral vs subcutaneous adipose tissue, their role in obesity and potential differential depot-specific correlation with anthropometric and metabolic traits. Here, we set out to describe **(i)** the gene expression profile of m6A writers, readers and erasers in visceral vs subcutaneous adipose tissue with a potential depot-specificity perspective; **(ii)** to analyze its relationship to clinically relevant traits of obesity in several well-described cohorts and **(iii)** to test whether SNP markers in m6A regulators associate with gene expression and clinical traits.

## Methods

### Study Populations

#### Adipose Tissue Leipzig Cohort

We have used data from the Adipose tissue Leipzig cohort (*N*=63, mean age 53 ± 16 years, mean BMI 36.1 ± 13.9 kg/m^2^) (25, 26), for which genome wide gene expression data are available for intra-individually paired samples of subcutaneous (SAT) and omental visceral adipose tissue (OVAT). Samples were taken at metabolic surgery or other procedures such as cholecystectomy as reported previously (25, 27). Patients were categorized based on BMI according to the WHO classification: lean ≥18; <25 kg/m²; overweight ≥25; <30 kg/m²; obese≥ 30 kg/m². This cohort comprises lean (*N*=23), overweight (*N*=3) and obese individuals (*N*=37). A range of anthropometric and metabolic variables available for this cohort were used in the here presented work. By using computed tomography measurements subjects with obesity were categorized in subcutaneous or visceral obese (CT-ratio represents the ratio of visceral/subcutaneous fat area). Another sample set of *N*=46 paired adipose tissue samples from SAT and OVAT from obese patients was used to support observed gene expression effects (10 samples overlapped with the initial Adipose tissue Leipzig cohort). RNA was extracted by using standard approaches (SIGMA ALDRICH, Saint Louis, USA and Qiagen, Hilden, Germany). RNA integrity and concentration were examined using Agilent 2100 Bioanalyzer (Agilent Technologies, CA, USA). RNA samples with RNA integrity values (RIN) of less than 5 were excluded from further analysis. All characteristics of the study populations are summarized in **Table 1**. The ethics committee of the University of Leipzig and the Regional Committee for Medical and Health Research Ethics for South Eastern Norway have approved all study protocols and written informed consents were collected from all study participants.

**Table 1:** Main characteristics of the study populations.

	Adipose tissue Leipzig cohort				Sorbs cohort			
				*N* per trait				*N* per trait
	Total	Lean	Obese	(total/lean/obese)	Total	Lean	Obese	(total/lean/obese)
***N***	63	23	37		1049	387	232	
**sex (m/f)**	16/47	7/16	7/30		418/617	114/273	86/146	
**T2D (yes/no)**	14/49	1/22	13/24		106/900	12/365	57/169	
**age (years)**	53 ± 16	65 ± 11	45 ± 13	63/23/37	48 ± 16	39 ± 15	58 ± 13	1035/387/232
**BMI (kg/m2)**	36.1 ± 13.9	22.8 ± 1.7	45.1 ± 11.2	63/23/37	26.9 ± 4.9	22.3 ± 1.7	34 ± 3.8	1022/387/232
**visceral fat area (cm²)**	164 ± 128	66 ± 88	252 ± 94	55/23/29	n.a.	n.a.	n.a.	n.a.
**subcutanoues fat area (cm²)**	583 ± 635	100 ± 232	1016 ± 570	55/23/29	n.a.	n.a.	n.a.	n.a.
**CT-ratio (vis./subc. fat area)**	0.62 ± 0.51	0.95 ± 0.59	0.33 ± 0.22	55/23/29	n.a.	n.a.	n.a.	n.a.
**Waist (cm)**	104 ± 30	78 ± 17	126 ± 21	54/22/29	90.9 ± 14	78 ± 8	107 ± 12	1012/377/230
**Hip (cm)**	109 ± 24	89 ± 10	126 ± 18	54/22/29	104 ± 9	97 ± 6	115 ± 9	1005/387/226
**Waist-to-hip ratio**	0.95 ± 0.15	0.88 ± 0.11	1.01 ± 0.15	54/22/29	0.87 ± 0.10	0.81 ± 0.08	0.93 ± 0.09	1002/376/226
**body fat %**	35.44 ± 13.87	21.66 ± 7.52	43.75 ± 9.87	43/14/27	21.45 ± 9.54	14.96 ± 5.36	32.86 ± 9.31	1008/380/226
**Fasting Plasma Glucose (mmol/l)**	5.86 ± 1.31	5.47 ± 1.01	6.16 ± 1.45	63/23/37	5.55 ± 1.20	5.06 ± 0.69	6.28 ± 1.64	1021/382/229
**Fasting Plasma Insulin (pmol/l)**	94.70 ± 141.1	22.64 ± 65.53	148.52 ± 158.75	32/5/26	42.39 ± 27.96	31.21 ± 18.55	62.96 ± 36.18	1025/385/228
**HbA1c %**	5.71 ± 0.66	5.28 ± 0.29	6.03 ± 0.67	61/23/35	n.a.	n.a.	n.a.	n.a.
**HDL Cholesterol (mmol/l)**	1.33 ± 0.39	1.6 ± 0.47	1.17 ± 0.25	47/16/28	1.63 ± 0.40	1.83 ± 0.39	1.49 ± 0.36	1006/377/226
**LDL Cholesterol (mmol/l)**	3.28 ± 1.29	2.78 ± 1.00	3.63 ± 1.39	48/17/28	3.37 ± 0.96	2.95 ± 0.91	3.56 ± 0.90	1006/377/226
**Triglycerids (mmol/l)**	1.19 ± 0.5	1.01 ± 0.28	1.37 ± 0.57	31/12/2017	1.31 ± 0.88	0.96 ± 0.53	1.72 ± 1.10	1006/377/226

All data are shown as mean ± S.D. (standard deviation). N, Number of subjects; T2D, diagnosed type 2 diabetes; BMI, body mass index (WHO classification: lean≥18; <25 kg/m²; <30 kg/m²obese≥ 30 kg/m²); n.a., not available; m, male; f, female.

#### Sorbs Cohort

In the present study a total of 1049 individuals from the Sorbs population, a German minority of Slavonic origin, were included (28). Of those, *N*=900 non-diabetic individuals (mean age 48 ± 16 and mean BMI 26.9 ± 4.9 kg/m^2^) were used for statistical analysis and a portfolio of clinical variables was available, including anthropometric and metabolic variables. The main study population characteristics are summarized in **Table 1**. The ethics committee of the University of Leipzig has approved the study protocols and written informed consents were collected from all study participants.

### Extraction of Gene Expression Data

mRNA expression data for all described m6A regulators in paired human adipose tissue samples were extracted for the Adipose tissue Leipzig cohort from our own work published previously elsewhere (25) for a total of *N*=63 with expression data available for SAT in *N*=56 and OVAT in *N*=48 (overlapping data in SAT and OVAT *N*=41). Further, mRNA expression data from peripheral blood mononuclear cells (PBMCs) for m6A regulators were available for the Sorbs cohort and were extracted from a genome wide data set for *N*=1049 subjects (29). Briefly, Illumina human HT-12 expression chips were used to generate mRNA expression data for both studies. Expression data were background-corrected, log-transformed and quantile-normalized. Differential expression analysis was performed using the R package oposSOM (30).

### Extraction of SNP Data

Genotype data of 41 single nucleotide polymorphisms (SNP) markers (24 SNPs in the *METTL3* locus; 17 SNPs in the *YTHDF3* locus) were extracted from a genome wide SNP data set available for 840 non-diabetic individuals from the German Sorbs population (31). All genotype data were analysed for being in Hardy-Weinberg equilibrium (HWE). We excluded one SNP marker in *METTL3* and five SNP markers in *YTHDF3* from further analyses because of being significantly different from HWE with p<0.05. Moreover, 14 SNP markers in *METTL3* and six SNPs in *YTHDF3* were in linkage disequilibrium (LD) and excluded from further analyses (**Supplementary Figure 1**).

### Expression Measurements by Using Reverse Transcriptase Quantitative PCR (RT-qPCR)

RT-qPCR was performed in triplicates on a Quantstudio 7 Flex system (ThermoFisher Scientific) using target specific primers (**Supplementary Table 1**) and PowerUp SYBR green (ThermoFisher Scientific). Gene expression levels were calculated by relative quantification with *PPIA* as housekeeping gene (2 ^(-CT gene of interest – CT^ *^PPIA^*)). Primers were designed to cover all main transcript variants of the genes and to cross exon-intron borders.

### Statistical Analyses

All statistical analyses were performed using SPSS statistics software 26 (SPSS, Inc. Chicago, IL) and GraphPad Prism 8 (GraphPad, San Diego, Ca, USA). Prior to analyses, data were tested for normal distribution by using both visual inspection of histograms and one-sample Kolmogorov-Smirnov test. Non-normally distributed variables were either logarithmically transformed to approximate normal distribution or non-parametric test were applied. Data are presented as mean ± standard deviation if not stated otherwise. Paired Student’s t-tests or Wilcoxon’s signed rank test were used to test for adipose tissue depot-specific gene expression whilst independent group statistics was used to test for differences between lean subjects and individuals with obesity. Linear regression analyses adjusted for age, gender and ln_BMI were performed to test for linear relationship between gene expression levels or SNPs and clinical variables (except for BMI in analyses where BMI was used as an independent variable). Genetic association analyses were performed for additive (mm vs Mm vs MM) or dominant (mm + Mm vs MM) mode of inheritance (m=minor allele; M=major allele). Different significance thresholds were applied to different sections of the work. For comparisons of means between lean and obese or SAT and OVAT, P<0.05 was used. When testing for correlations with clinical variables in two adipose tissue depots Bonferroni correction was used to take into account multiple testing (0.05/6 (highly correlated traits were reduced to three x 2 tissue depots=number of tests=6)). We lowered the study-specific significance threshold for these tests in adipose tissue to *P*=0.0083. All P-values > 0.0083 but < 0.05 were considered to be of nominal statistical significance. For genetic association analyses in the Sorbs cohort, we also applied Bonferroni correction taking into account clinical traits (*N*=3 after aggregating highly correlated traits) and the number of SNP markers tested (*N*=15). We lowered the study-specific significance threshold for this part of the analyses to *P*=0.0011 (0.05/45). All P-values > 0.0011 but < 0.05 were considered to be of nominal statistical significance. All P-values given are uncorrected for multiple testing.

## Results

### Expression of m6A Writers, Erasers and Readers in Human Adipose Tissue Differs Between Lean and Obese and Correlates With Clinical Variables

To evaluate the impact of gene expression level of m6A regulators on clinically relevant traits of obesity, we first tested whether m6A writers, erasers, and readers are significantly different expressed between individuals with obesity and lean subjects.

### Gene Expression of m6A Writers, Erasers and Readers Correlates with Obesity

From our previously published genome-wide data set (Keller M et al., 2016), we extracted paired gene expression data from OVAT and SAT for 11 m6A regulators (**Table 2** and **Supplementary Figure 2**). In OVAT, we identified by using independent t-tests or Mann-Whitney-U tests, the writers *WTAP* (ILMN_2279339; *P*=0.011) and *VIRMA* (*P*=0.028) as differentially expressed between lean and obese (**Table 2**). Further, we found the eraser *ALKBH5* (*P*=0.023, **Table 2**) as well as the readers *YTHDF1* (*P*=0.007), and *YTHDF2* (*P*=0.045) differentially expressed between lean and obese. The most significant differences between the two groups were, however, found in SAT at the writer *VIRMA* (*P*=0.005) and the reader *YTHDC1* (ILMN_1707506; *P*=9.26x10^-5^), both being significantly lower expressed in individuals with obesity. All data are summarized in **Table 2**. No significant relationship of gene expression of m6A regulators with T2D was observed (data not shown).

**Table 2:** Comparison of mean gene expression of m6A regulators between lean and obese in adipose tissue.

Gene	Probe	Depot	N	Lean		Obese		p-value
			Lean/Obese	Mean	SD of Mean	Mean	SD of Mean	
**m6A writers**								
METTL3	ILMN_1655635	SAT	20/34	-0.0135	0.0588	-0.0116	0.0619	n.s.^2^
		OVAT	15/31	0.0022	0.06	0.0246	0.1103	n.s.^2^
METTL14	ILMN_22124523	SAT	20/34	0.0004	0.061	-0.0029	0.0423	n.s.^1^
		OVAT	15/31	0.0035	0.0498	0.0048	0.0512	n.s.^1^
WTAP	1: ILMN_2279339	SAT	20/34	0.0126	0.1093	0.0263	0.1193	n.s.^2^
		OVAT	15/31	-0.0839	0.0807	0.0063	0.1082	**0.011^2^**
	2: ILMN_2260725	SAT	20/34	-0.0046	0.0576	0.0078	0.0565	n.s.^1^
		OVAT	15/31	-0.0151	0.083	0.0017	0.0708	n.s.^1^
	3: ILMN_2356559	SAT	20/34	-0.0196	0.0649	0.0044	0.0765	n.s.^1^
		OVAT	15/31	-0.0102	0.0577	-0.0033	0.0558	n.s.^1^
	4: ILMN_1734544	SAT	20/34	0.0165	0.0911	0.0116	0.0818	n.s.^2^
		OVAT	15/31	-0.0167	0.1356	-0.0182	0.065	n.s.^2^
	5: ILMN_1657618	SAT	20/33	0.0403	0.1046	-0.0177	0.0805	**0.041^1^**
		OVAT	15/31	0.032	0.078	-0.0025	0.0944	n.s.1
VIRMA	ILMN_1813635	SAT	20/34	0.0568	0.0808	-0.0059	0.0621	**0.005^1^**
		OVAT	15/31	0.0147	0.0705	-0.0343	0.0582	**0.028^1^**
**m6A erasers**								
FTO	ILMN_2288070	SAT	20/34	0.0212	0.0513	0.0311	0.0637	n.s.^1^
		OVAT	15/31	-0.0311	0.0717	-0.0285	0.0637	n.s.^1^
ALKBH5	ILMN_1657283	SAT	20/34	-0.0213	0.0483	0.0083	0.0356	**0.021^2^**
		OVAT	15/31	-0.0179	0.0399	0.0113	0.0385	**0.023^2^**
**m6A readers**								
YTHDF1	ILMN_1753885	SAT	20/34	0.0046	0.0546	-0.0082	0.0398	n.s.^2^
		OVAT	15/30	-0.025	0.0468	0.015	0.049	**0.007^2^**
YTHDF2	ILMN_1730658	SAT	20/34	-0.0163	0.0487	0.0112	0.0659	n.s.^1^
		OVAT	15/31	-0.0293	0.0546	0.0071	0.0569	**0.045^1^**
YTHDF3	ILMN_1657470	SAT	20/34	-0.0078	0.0518	0.0013	0.0577	n.s.^1^
		OVAT	15/31	-0.0031	0.0405	0.0037	0.0649	n.s.^1^
YTHDC1	1: ILMN_1666111	SAT	20/34	-0.008	0.0674	0.0201	0.0777	n.s.^1^
		OVAT	15/31	-0.019	0.1035	-0.0133	0.0763	n.s.^1^
	2: ILMN_1670878	SAT	20/34	-0.0049	0.074	0.009	0.0664	n.s.^1^
		OVAT	15/31	0.0153	0.0613	-0.0133	0.0684	n.s.^1^
	3: ILMN_1707506	SAT	20/34	0.0403	0.0647	-0.0359	0.055	**9.262x10^-5 1^**
		OVAT	15/31	0.0133	0.0631	0.0016	0.0782	n.s.^1^
IGF2BP2	ILMN_1702447	SAT	20/34	-0.0082	0.1097	0.0009	0.1306	n.s.^1^
		OVAT	15/31	-0.0313	0.1112	0.005	0.1637	n.s.^1^

P-values were calculated using 1) independent t-test for normally distributed variables and 2) Mann-Whitney-U test for not normally distributed variables; SAT, Subcutaneous adipose tissue; OVAT, Omental visceral adipose tissue; SD, Standard deviation; n.s., not significant.

P-values < 0.05 are highlighted in bold.

### Gene Expression Correlates Differentially With Measures of Obesity and Fat Distribution

**Figure 1:**
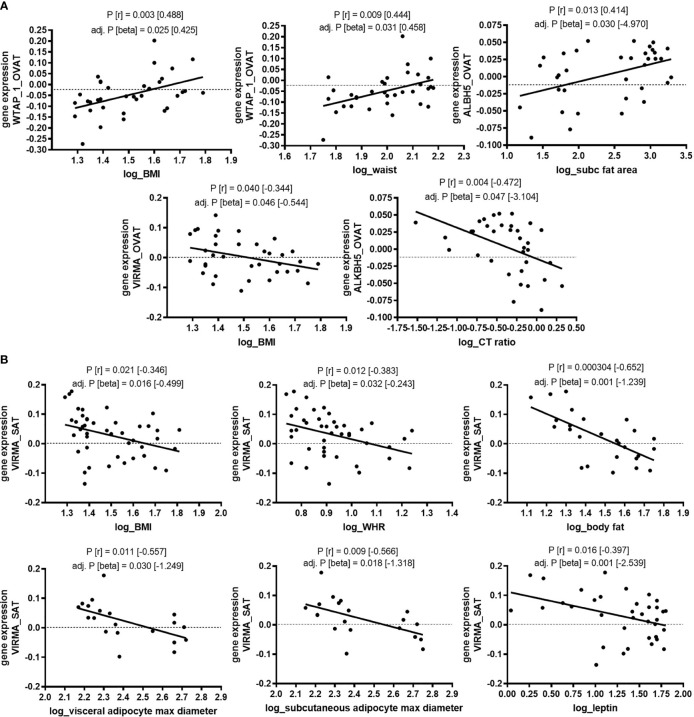
Association of gene expression with clinical variables of obesity and fat distribution in non-diabetic subjects (adipose tissue Leipzig cohort). Correlation of gene expression (log transformed, normalized intensities) with clinical variables of obesity and fat distribution in non-diabetic subjects. **(A)** in OVAT and **(B)** in SAT. *P*-values were calculated by using bivariate Pearson or Spearman correlation. Significant correlation results are shown for correlations that survive adjustment for age, and sex in linear regression analysis. BMI, body mass index; WHR, waist to hip ratio; OVAT, omental visceral adipose tissue; SAT, subcutaneous adipose tissue; Beta, non-standardized regression coefficient from linear regression; *P*=*P*-value; r, correlation coefficient from bivariate correlation.

The statistically significant association between gene expression of a number of m6A regulators and obesity led us to hypothesize that gene expression of these regulators correlates also with continuous traits related to obesity and fat distribution. Indeed, among non-diabetic individuals we find that gene expression of *WTAP* in OVAT correlates with body mass index (BMI) (*P*=0.003) and waist circumference (*P*=0.009). In line with this, expression of *VIRMA*in OVAT, which associates with obesity, shows a negative correlation with BMI (*P*=0.040). The expression of the eraser *ALKBH5* in OVAT shows a nominal significant correlation with the amount of subcutaneous fat area (*P*=0.013) as well as the ratio of visceral to subcutaneous fat area (CT-ratio, *P*=0.004). All these bivariate correlations withstand adjustment for age and sex in linear regression analyses (all adjusted *P*-values are shown in **Figure 1A**).

In line with reduced gene expression of *VIRMA* in patients with obesity, we find in SAT gene expression of *VIRMA* correlated to BMI (*P*=0.021), waist to hip ratio (WHR) (*P*=0.012), percentage of body fat (*P*=0.0003), maximal adipocyte diameter in SAT (*P*=0.009) and OVAT (*P*=0.011), and leptin serum levels (*P*=0.016). Further, to substantiate evidence for a linear relationship, we performed linear regression analysis adjusted for covariates such as age and sex. All described correlations are also significant after such adjustment. All adjusted *P*-values are shown in **Figure 1B**.

These data indicate that expression of certain m6A regulators in adipose tissue is associated with obesity and a range of continuous clinical variables related to obesity and fat distribution. However, this seems also dependent on the respective adipose tissue depot. Therefore, we next tested the hypothesis that the expression of m6A regulators may be adipose tissue depot-specific.

### Inter-Depot Specific Adipose Tissue Expression Between SAT and OVAT

**Figure 2:**
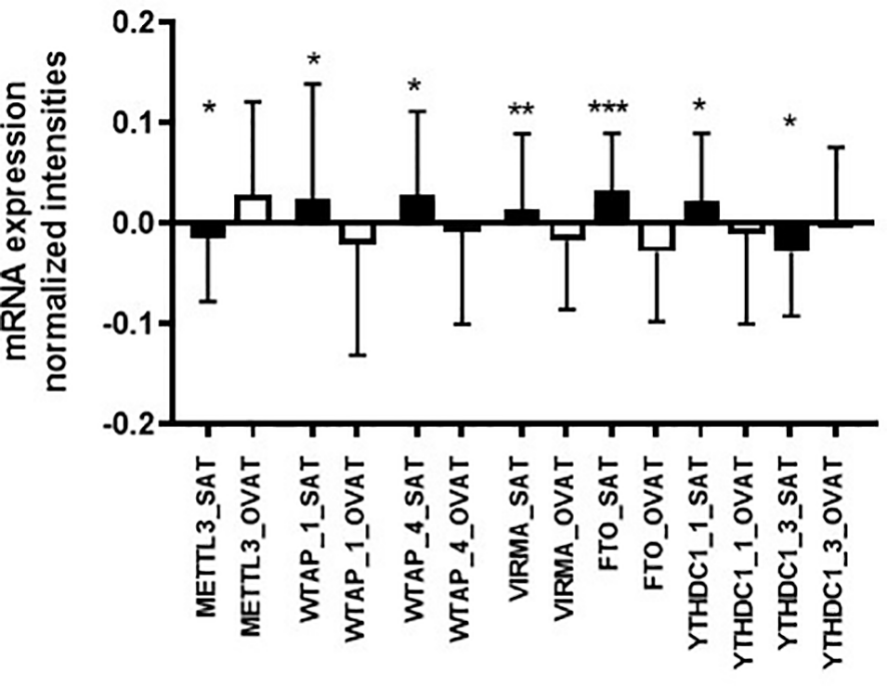
Adipose tissue depot-specific differences in gene expression of m6A writers, reader and erasers (adipose tissue Leipzig cohort). Differences in gene expression (log transformed, normalized intensities) between SAT and OVAT. Data are presented as mean ± SD. *P*-values were calculated using paired samples t-tests or Wilcoxon signed rank test in n=41 and given as asterix (*P < 0.05; **P < 0.01; ***P < 0.001). OVAT, omental visceral adipose tissue; SAT, subcutaneous adipose tissue. The following Illumina probes are presented: METTL3=ILMN_1655635; WTAP_1=ILMN_2279339; WTAP_4=ILMN_1734544; VIRMA=ILMN_1813635; FTO=ILMN_2288070; YTHDC1_1=ILMN_1666111; YTHDC1_3=ILMN_1707506.

By using paired statistics, we observed that gene expression of a number of m6A regulators is significantly different between OVAT and SAT (**Table 3**). We show that the writers *METTL3* (*P*=0.014), *WTAP* (probe ILMN_2279339 *P*=0.031; and probe ILMN_1734544 *P*=0.022) and *VIRMA* (*P*=0.002) confer significant inter-depot specificity, with highest significance for *VIRMA* (**Table 3**). Moreover, highly significant differences were identified for the eraser *FTO* (*P*=2.257x10^-4^), the strongest obesity candidate gene identified in genetic studies (19). Further, two probes targeting the reader *YTHDC1* (probe ILMN_1666111 *P*=0.047; and probe ILMN_1707506 *P*=0.045) showed borderline significance between the two adipose tissue depots. All data are summarized in **Table 3**. Among those five m6A regulator genes exhibiting significant depot specificity, the gene encoding the catalytic subunit of the m6A methyltransferase complex, *METTL3*, shows higher expression in OVAT whilst other subunits of the methyltransferase complex *WTAP* and *VIRMA* show higher expression in SAT. *FTO* is significantly higher expressed in SAT confirming previous data (32) whilst we found gene expression for two different probes targeting the reader *YTHDC1* either increased or decreased in OVAT. All data are summarized in **Figure 2**.

**Table 3:** Differential gene expression of m6A regulators between SAT and OVAT.

Gene	Probe	N	SAT		OVAT		p-value
			Mean	SD of Mean	Mean	SD of mean	
**m6A writers**							
METTL3	ILMN_1655635	41	-0.01387	0.063791	0.02829	0.093343	**0.014^b^**
METTL14	ILMN_22124523	41	0.0006	0.046264	0.00673	0.051285	n.s^a^
WTAP	1: ILMN_2279339	41	0.02405	0.115028	-0.02073	0.110101	**0.031^b^**
	2: ILMN_2260725	41	0.00559	0.053769	-0.00879	0.071324	n.s^a^
	3: ILMN_2356559	41	0.0044	0.075411	-0.00279	0.054819	n.s^a^
	4: ILMN_1734544	41	0.02897	0.082764	-0.0077	0.09224	**0.022^b^**
	5: ILMN_1657618	40	-0.01555	0.071808	0.00402	0.084863	n.s^a^
VIRMA	ILMN_1813635	41	0.01375	0.076048	-0.0173	0.067723	**0.002^a^**
**m6A erasers**							
FTO	ILMN_2288070	41	0.03308	0.05702	-0.02831	0.068946	**2.257x10^-4 a^**
ALKBH5	ILMN_1657283	41	0.00433	0.038695	0.00376	0.041619	n.s^b^
**m6A readers**							
YTHDF1	ILMN_1753885	40	-0.00949	0.04035	0.00177	0.050554	n.s^b^
YTHDF2	ILMN_1730658	41	0.01221	0.057429	-0.00354	0.057748	n.s^a^
YTHDF3	ILMN_1657470	41	0.0049	0.0515	0.0062	0.05516	n.s^a^
YTHDC1	1: ILMN_1666111	41	0.02307	0.067122	-0.00951	0.08985	**0.047^a^**
	2: ILMN_1670878	41	0.005	0.066725	-0.00409	0.070425	n.s^a^
	3: ILMN_1707506	41	-0.02831	0.063662	0.00131	0.07475	**0.045^a^**
IGF2BP2	ILMN_1702447	41	-0.01219	0.122958	-0.00586	0.152985	n.s^a^

P-values were calculated using a) paired t-tests for normally distributed variable and b) Wilcoxon signed rank test for non-normally distributed variables. WHO classification: lean ≥18; <25 kg/m²; overweight ≥25; <30 kg/m²; obese≥ 30 kg/m²; N (lean/overweight/obese)=12/1/28; SAT, Subcutaneous adipose tissue; OVAT, Omental visceral adipose tissue; SD, Standard deviation; n.s., not significant.

P-values < 0.05 are highlighted in bold.

**Figure 3:**
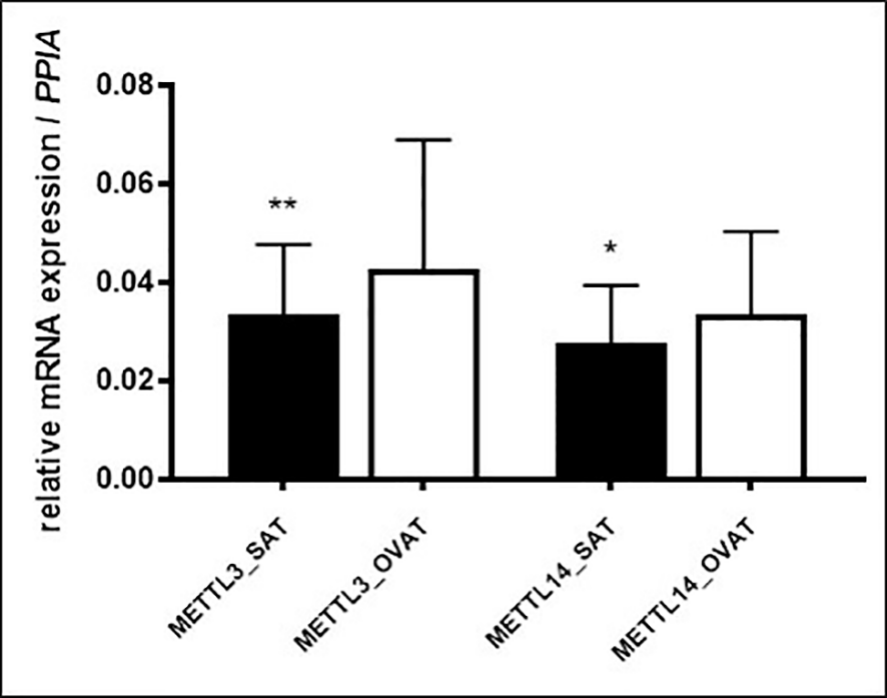
m6A writers showing significant differential gene expression in SAT vs. OVAT (RT-qPCR validation). Differences in gene expression between SAT and OVAT measured by RT-qPCR. Relative expression values using *PPIA* as housekeeping gene. Data are presented as mean ± SD. *P*-values were calculated using Wilcoxon signed rank test in n=46 individuals. (*P < 0.05; **P < 0.01). OVAT, omental visceral adipose tissue; SAT, subcutaneous adipose tissue.

To verify these results, we performed RT-qPCR in another sample set (*N*=46) of intra-individually paired human adipose tissue samples of OVAT and SAT. Gene expression was measured for the following genes: writers: *METTL3*, *METTL14*, *WTAP* and *VIRMA*, erasers: *FTO* and *ALKBH5* and readers: *YTHDF1*, *YTHDF2*, *YTHDC1* and *IGF2BP2*. Interestingly, we confirm our finding of inter-depot specific expression for *METTL3* (*P*=0.001, **Figure 3**) showing the same effect direction with higher expression in OVAT. In addition, we observed increased OVAT expression of the second main component of the methyltransferase complex *METTL14* (*P*=0.012, **Figure 3**). No other differences were identified in this cohort. All data are summarized in **Table 4**.

**Table 4:** Differential gene expression of m6A writers, erasers and readers between SAT and OVAT measured by RT-qPCR.

Gene	N	SAT		OVAT		p-value
		Mean	SD of Mean	Mean	SD of Mean	
**m6A writers**						
METTL3	46	0.0333	0.01434	0.0427	0.02628	**0.001**
METTL14	46	0.0277	0.01165	0.0335	0.01681	**0.012**
WTAP	46	0.1485	0.12453	0.1218	0.07448	n.s
VIRMA	46	0.0527	0.02732	0.0542	0.02878	n.s
**m6A erasers**						
FTO	46	0.0705	0.0724	0.0769	0.07478	n.s
ALKBH5	46	0.0818	0.05545	0.0852	0.05669	n.s
**m6A readers**						
YTHDF1	46	0.0286	0.01545	0.0273	0.01374	n.s
YTHDF2	46	0.1382	0.16016	0.1298	0.11158	n.s
YTHDC1	46	0.0656	0.03214	0.0696	0.03237	n.s
IGF2BP2	46	0.0318	0.01897	0.0275	0.01739	n.s

P-values were calculated using Wilcoxon signed rank test for non-normally distributed variables. WHO classification: lean ≥18; <25 kg/m²; overweight ≥25; <30 kg/m²; obese≥ 30 kg/m²; SD, Standard deviation; SAT, Subcutaneous adipose tissue; OVAT, Omental visceral adipose tissue; n.s., not significant.

P-values < 0.05 are highlighted in bold.

### Gene Expression of m6A Erasers and Readers in PBMCs Correlates With Obesity

**Figure 4:**
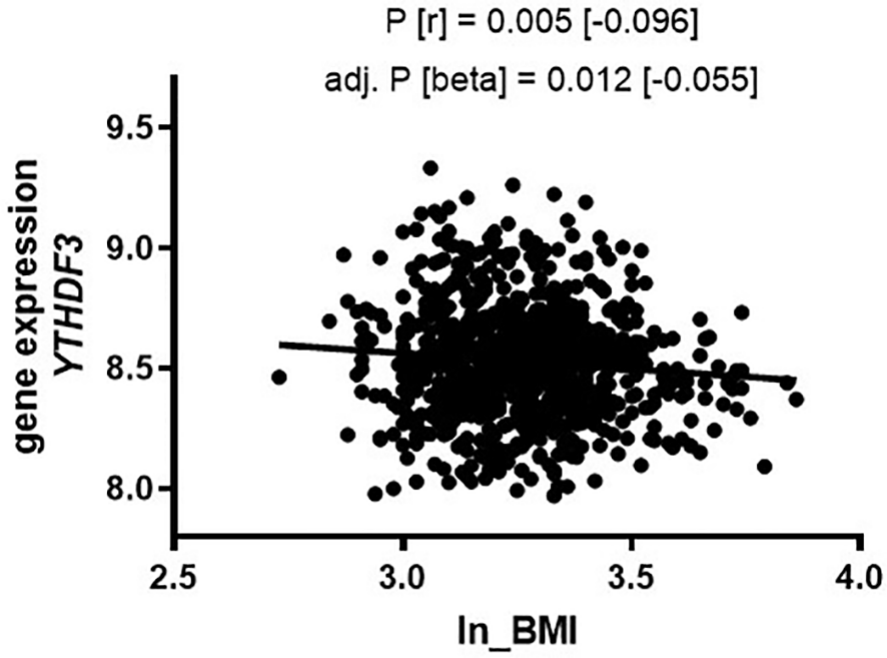
Gene expression of *YTHDF3* (PBMCs) in the Sorbs cohort correlates with BMI. Correlation of gene expression with BMI in the Sorbs population in non-diabetic subjects (*N*=875). *P*-values were calculated by using bivariate Spearman correlation (top *P*-value) and linear regression analysis for ln_BMI adjusted for age and sex (bottom line). BMI, body mass index; Beta=non-standardized regression coefficient from linear regression; *P*=*P*-value, r=correlation coefficient from bivariate correlation.

Based on our data from adipose tissue showing that gene expression of several m6A regulators correlates with obesity and clinical variables, we hypothesized that gene expression of m6A writers, readers and erasers in PBMCs might also be different between lean subjects and individuals with obesity. Therefore, we extracted gene expression data for m6A regulators from our existing genome wide data set (29) and tested for differences of the mean expression values between lean and obese by using independent t-test statistics. Our data show that none of the covered probes for the writers are different between the two groups (**Table 5**). However, we find a strong difference for the eraser *ALKBH5* showing higher expression in obese (*P*=5.80x10^-5^), as well as for the two readers *YTHDF1* (*P*=0.049) and *YTHDF3* (*P*=0.003) both showing higher expression in lean individuals. To better understand whether the observed differences of means reflect an association of gene expression with BMI, we performed linear regression analysis adjusted for age and sex as additional confounders. These analyses resulted for *YTHDF3* in a *P*=0.012 (beta -0.055; [-0.098; -0.012]), whilst no nominal significance was found for *ALKBH5* and *YTHDF1*. **Figure 4** illustrates the negative linear relationship between *YTHDF3* expression and BMI. No significant association of gene expression of m6A regulators with T2D was observed (data not shown).

**Table 5:** Comparison of mean gene expression of m6A regulators between lean and obese in the Sorbs cohort.

Gene	Probe	N	Lean		Obese		p-value
		Lean/Obese	Mean	SD of Mean	Mean	SD of Mean	
**m6A writers**							
METTL3	ILMN_1655635	382/227	8.740326	0.3575872	8.73174	0.3787329	n.s
METTL14	ILMN_22124523	382/227	7.144649	0.2171319	7.14253	0.2632073	n.s
WTAP	1: ILMN_2279339	382/227	9.370565	0.2706417	9.33891	0.254263	n.s
	2: ILMN_2260725	382/227	6.527478	0.2282255	6.52325	0.2277159	n.s
	3: ILMN_2356559	382/227	6.881981	0.3160953	6.92075	0.312121	n.s
	4: ILMN_1734544	382/227	6.470573	0.2863549	6.46303	0.2961954	n.s
	5: ILMN_1657618	382/227	6.526426	0.2998339	6.47908	0.2864933	n.s
**m6A erasers**							
FTO	ILMN_2288070	382/227	7.49235	0.2440247	7.48991	0.2792386	n.s
ALKBH5	ILMN_1657283	382/227	12.00184	0.1602151	12.0582	0.1688926	**5.80x10^-5^**
**m6A readers**							
YTHDF1	ILMN_1753885	382/227	9.568409	0.1794507	9.5409	0.1582593	**0.049**
YTHDF2	ILMN_1730658	382/227	9.570496	0.2648845	9.58526	0.2376172	n.s
YTHDF3	ILMN_1657470	382/226	8.548154	0.2412513	8.49755	0.1997413	**0.005**
YTHDC1	1: ILMN_1666111	382/227	6.836997	0.4142519	6.86563	0.4287415	n.s
	2: ILMN_1670878	382/227	5.974073	0.1735351	5.98633	0.1975569	n.s
	3: ILMN_1707506	382/227	11.429733	0.2175373	11.4067	0.222591	n.s
IGF2BP2	ILMN_1702447	382/227	8.156624	0.3614102	8.15192	0.3238712	n.s

P-values were calculated using independent t-test for normally distributed variables. WHO classification: lean ≥18; <25 kg/m²; overweight ≥25; <30 kg/m²; obese≥ 30 kg/m²; SD, Standard deviation; n.s., not significant. P-values < 0.05 are highlighted in bold.

### Genetic Variants in *METTL3* and *YTHDF3* Associate With Anthropometric and Metabolic Variables Related to Obesity

**Figure 5:**
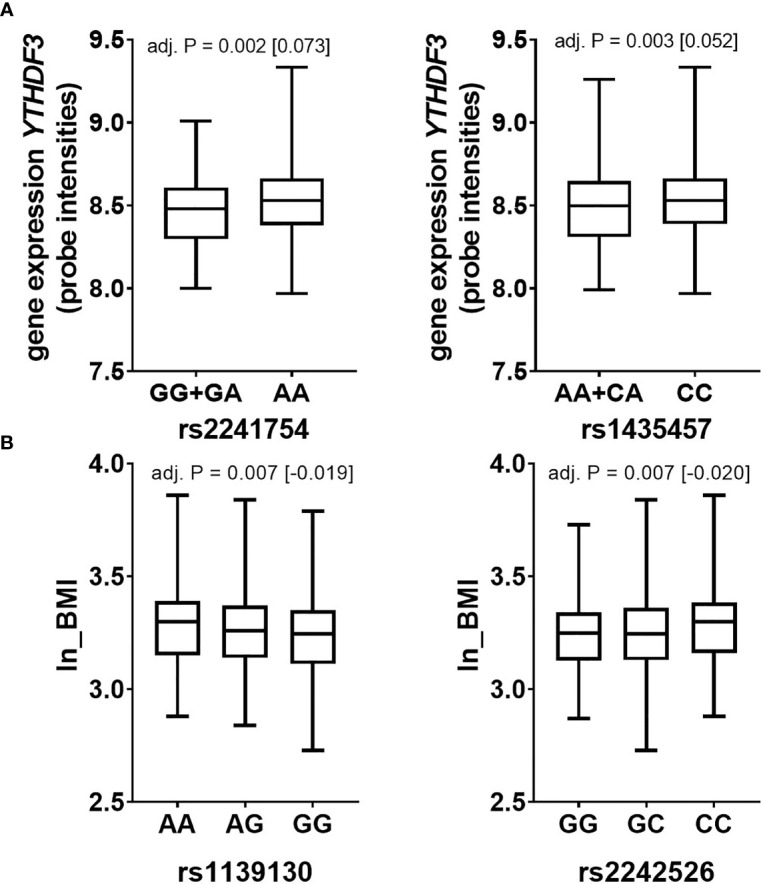
Genetic variation in *YTHDF3* and *METTL3* associates with expression and BMI respectively (Sorbs cohort). Data are presented as boxplots with median and quartile distribution for SNPs in **(A)** *YTHDF3* and **(B)** *METTL3*. *P*-values were calculated by using linear regression analyses adjusted for age, sex and BMI (except for ln_BMI) by using an additive or dominant mode of inheritance. Numbers of subjects per SNP are as following: *METTL3*: rs1139130 AA=209, AG=390, GG=241; rs2242526 GG=151, GC=415, CC=274; *YTHDF3*: rs2241754 AA=733, GG+GA= 107; rs1435457 CC=586, AA+AC=254. *P*=*P*-value; BMI-body mass index.

Our data have shown so far that **(i)** *METTL3* expression is adipose tissue depot-specific with higher expression levels in OVAT in two sample sets and, **(ii)** that *YTHDF3* gene expression in PBMCs differs between lean and obese and is negatively associated with BMI. To test the hypothesis that the observed effects may be at interplay with genetic variation, we analysed genotypes of 15 SNP markers (9 SNPs for *METTL3* and 6 SNPs for *YTHDF3*) extracted from our existing genome wide data set (31). We performed genetic association analyses of these markers in *N*=840 non-diabetic individuals with anthropometric and metabolic variables available in the Sorbs (**Table 1**). We identified two SNP markers in the locus of the m6A reader *YTHDF3* (rs2241754 upstream variant, and rs1435457 intronic variant) correlating with *YTHDF3* gene expression. Carriers of the minor G allele of rs2241754 show nominal significantly lower expression levels compared to carriers of the major A allele (**Figure 5A**). Similar results were obtained for rs1435457 with decreased expression levels in minor A allele carriers (**Figure 5A**). Further, we observed that two SNP markers in *METTL3* (rs1139130 intragenic coding and rs2242526 intronic variant) associate with BMI (**Figure 5B**), though these effects were not strong enough to withstand logistic regression analysis for obesity adjusted for age and sex (data not shown). These results imply that genetic variation in *METTL3* and *YTHDF3* is related to either BMI or gene expression which may potentially modulate a relationship between gene expression of m6A regulators and variables of obesity and fat distribution.

## Discussion

In this work, we studied the gene expression profiles of major players of the m6A writer complex, several readers and the two known erasers in human adipose tissue and in PBMCs from different cohorts. This is the first report analysing m6A regulators in paired human adipose tissue biopsies (SAT and OVAT) and our results show, that **(i)** gene expression of some m6A regulators correlates with obesity and clinical variables; **(ii)** several regulators are differentially expressed between the two adipose tissue depots and **(iii)** genetic variation in selected regulators is related to both gene expression levels and BMI.

### m6A Regulators in Adipose Tissue Correlate With Obesity and Clinical Variables

By comparing gene expression of m6A regulators in adipose tissue between individuals with obesity and lean controls, we identify several genes that associate with obesity in SAT and/or OVAT. These include the writers *WTAP* and *VIRMA*, the eraser *ALKBH5*, and genes encoding reader proteins such as *YTHDF1, YTHDF2* and *YTHDC1*. These results imply a relationship of gene expression with weight and prompted us to further test for a correlation with clinical variables related to obesity. We observed significant correlations with clinical parameters for *VIRMA, WTAP* and *ALKBH5*.

*VIRMA*, (also known as *KIAA1429*) encodes a subunit of the m6A methyltransferase complex and is together with WTAP, METTL3 and METTL14, required for its methyltransferase activity (33). VIRMA acts as a scaffolding protein, guiding region-selective methylation by recruiting the catalytic core components METTL3/METTL14/WTAP to stop codons and 3’UTR regions, influencing alternative polyadenylation and splicing (34). VIRMA is dysregulated in several cancer types, influencing cell proliferation in both a m6A-dependent and a m6A-independent manner (35). Interestingly, we find that gene expression of *VIRMA* associates with obesity both in SAT and OVAT showing lower expression in obesity. In line with this, expression of *VIRMA* negatively correlates with BMI both in SAT and VAT. Moreover, SAT expression of *VIRMA* is consistently negatively correlated with WHR, body fat percentage, adipocyte diameter and leptin levels, all being clinical variables related to fat distribution and obesity. Collectively, these data imply that altered levels of this writer complex subunit, which may contribute to altered m6A deposition, is involved in obesity. To our knowledge, so far nothing is known regarding VIRMA in the context of adipose tissue and metabolic dysfunction in obesity. Our results suggest a role for VIRMA in adipose tissue biology and metabolism, particularly in SAT, and further functional studies are required to investigate the role of this m6A writer in obesity.

WTAP is another main subunit of the m6A writer complex (36). In adipose tissue originating from omental visceral depots *WTAP* expression is higher among individuals with obesity. In line with this, we also find a positive correlation of *WTAP* expression in OVAT with BMI and waist circumference implying a depot-specific role in obesity. Indeed, *WTAP* is differentially expressed between the two depots. However, among the 5 probes available in our dataset such correlations were only found for one probe (ILMN_2279339). In support of our data, WTAP has been shown to have an essential role in adipogenic differentiation in mouse 3T3-L1 preadipocytes (17), and may be involved in adipose tissue dysfunction observed in morbidly obese individuals.

*ALKBH5* gene encodes one of the two known m6A demethylases and is reportedly involved in multiple biological mechanisms related to cancer such as proliferation (37) or metastasis (38). However, no direct role in metabolic diseases such as obesity is described so far. In adipose tissue, we find that subjects with obesity exhibit higher gene expression of *ALKBH5* in both fat depots compared to lean individuals. Further, expression in OVAT is positively correlated with subcutaneous fat area and negatively related to CT-ratio, both measures of fat distribution. These data imply a potential role of ALKBH5 in obesity and its clinical variables, which warrants further functional studies.

Collectively, our results add to and support previously published data from porcine models, demonstrating that m6A levels associate with fat mass and are involved in adipogenesis and lipid metabolism, suggesting a possible role for m6A writers in obesity (39). No significant relationship of gene expression of m6A regulators with T2D was observed in our data set.

### Adipose Tissue Depot-Specific Gene Expression of Major m6A Regulators

By comparing gene expression data from intra-individually paired adipose tissue depots, we find that multiple m6A regulators are differentially expressed between SAT and OVAT. Particularly, genes encoding the m6A writers show depot specific expression, with significant differences in almost all examined target genes. *METTL3*, *WTAP* (two out of 5 probes: ILMN_2279339 and ILMN_1734544) and *VIRMA* are all differentially expressed between SAT and OVAT in the microarray dataset. Depot specific gene expression of *METTL3* was confirmed in another sample set of adipose tissue analyzed by RT-qPCR. Moreover, we find also for *METTL14* a higher expression in OVAT compared to SAT. Several studies have reported an important role for m6A writer proteins in adipogenesis. METTL3, METTL14 and WTAP have been shown to be required for adipogenesis in mouse pre-adipocytes regulating mitotic clonal expansion (17, 40). Contrary to these data, adipogenic differentiation of porcine bone marrow derived stem cells is inhibited *via* METTL3 (41). Although contradictory results have been reported, results point towards an important role of m6A writers in metabolic disease and in adipogenesis, a role that may be dependent on species, fat depot or on the cellular context. Moreover, expression of *METTL3*, *METTL14*, and *WTAP* was reported to be increased in blood from patients with type 2 diabetes (T2D) (42), whilst METTL3 was reported to increase hepatic lipid accumulation through YTHDF2 dependent stabilization of PPARα (43). No evidence for differential gene expression in adipose tissue of *VIRMA* was previously reported, and collectively, our data indicate that inter-depot specific gene expression of m6A writers is involved in obesity.

In addition to writers, we also identified depot specific expression of the m6A eraser *FTO*, with higher expression in SAT, in line with previously published data (32) and data from the Genotype-Tissue Expression (GTEx) consortium (https://gtexportal.org). FTO-dependent de-methylation is reported to be essential for adipogenic differentiation (21), suggesting that depot specific expression may be involved in the reduced adipogenic capacity of OVAT (44). Further, the reader *YTHDC1* is also differentially expressed between SAT and OVAT, showing differential expression in 2 out of 3 probes, however with different effect directions between the two probes (ILMN_1666111: higher expression in SAT, ILMN_1707506: higher expression in OVAT). As the probes target different transcripts of *YTHDC1*, these results may suggest that different transcript variants and alternative splicing of this gene may contribute to depot-specific differences. Further transcript variant specific studies are needed to validate potential depot specific expression of *YTHDC1* isoforms. YTHDC1 is located in the cell nucleus, where it is involved in the nuclear export of m6A modified mRNAs as well as in m6A guided regulation of chromatin organization (45, 46). Differential expression of *YTHDC1* may, therefore, potentially also influence chromatin organization in an adipose tissue depot-specific manner, adding another level of complexity to gene regulation by m6A.

Taken together, these results indicate a depot specific role of m6A regulators, potentially introducing inter-depot-specific variability in mRNA metabolism. Such variability may potentially impact on adipogenesis, adipose tissue expansion capacity or metabolism, reflected by its correlation with clinical variables. Whether the observed expression differences have such functional consequences, either by targeting specific transcripts in the different tissues or by inducing global differences in m6A deposition remains to be investigated.

### The Role of m6A Regulators in Peripheral Blood Mononuclear Cells

In line with expression differences in adipose tissue between lean and obese individuals, we also found expression of m6A regulators associated with obesity in a dataset from PBMCs from the Sorbs population. In PBMCs, the eraser *ALKBH5* and the readers *YTHDF1* and *YTHDF3* associate with obesity. *ALKBH5* shows higher gene expression among individuals with obesity compared to lean subjects which is in line with our results from adipose tissue and thus, adding weight to a potential implication of *ALKBH5* in obesity. YTHDF3 is together with YTHDF1 and YTHDF2 a cytoplasmic reader protein facilitating transcript turnover and translation (47). In contrast to our data from adipose tissue, we do not find similar effect directions for these reader proteins in PBMCs. However, in line with an association with obesity, expression of *YTHDF3* is correlated to BMI. Altered gene expression of reader proteins in obesity may induce tissue specific functional consequences of m6A methylation and thus on RNA metabolism. However, no significant association of gene expression of any m6A regulator with T2D as a potential consequence of obesity was observed and functional studies are warranted to further evaluate potential biological consequences on RNA metabolism.

Taken together, these results show that expression of a number of m6A regulators is associated with obesity, both in adipose tissue and in PBMCs, suggesting that the m6A machinery is altered in obesity. However, what genes are affected appears to be tissue type dependent. Mapping of m6A modified transcripts and characterization of m6A readers and functional studies are required to further elucidate consequences of the observed differences.

### Genetic Variation in m6A Regulators

*METTL3* is differentially expressed between SAT and OVAT, whilst *YTHDF3* associates with obesity and BMI in blood. Thus, we then performed genetic analyses of these selected m6A regulators in the Sorbs. We observe a correlation of two SNP markers in the *YTHDF3* locus with its gene expression level suggesting that genetic variation underlying this reader protein may further impact on its potential role in obesity. However, no further relationships with any other clinical variables are observed and further genetic studies are needed to support or reject our results. Two SNP markers within the *METTL3* gene correlate with BMI implying that genetic variability in *METTL3* encoding the catalytic subunit of the m6A methyltransferase complex may influence clinical variables of obesity. No other clinical variables are related to the two SNPs. Our genetic data reveal a potential relationship of genetic variation in genes encoding m6A regulators with either clinical variables or gene expression. Whether such genetic variability impacts on the function of m6A regulators remains to be elucidated.

### Limitations

This is to our knowledge the first study analyzing gene expression of m6A regulators in paired samples from different human adipose tissue depots. Further, to support our findings we have included another cohort for which both gene expression data from PBMCs and genetic data were available. Despite these strengths, we are well aware of several limitations at different aspects in our study. First, adipose tissue is a heterogeneous tissue consisting of many cell types other than adipocytes. We performed our analyses on whole adipose tissue and potential effects from cell type composition cannot be either excluded or estimated. No data are available to account for cell type differences or blood cell count by using bioinformatic approaches. Further studies measuring expression in purified adipocytes or performing single cell gene expression profiling are warranted to provide deeper insights into the contribution of different cell types in the tissue. Second, the cohorts used in this study are relatively small which may have led to false positive or false negative results. The effect sizes of the observed differences are small and further studies are required to elucidate whether results from this study are of biological significance. Therefore, the results presented here need to be interpreted with caution to avoid overestimating the observed effects.

### Conclusion

Collectively, our data show that expression of m6A regulators is adipose tissue depot-specific and differentially related to clinical traits. We further show that several m6A regulators are associated with obesity. Taken together, these results point towards a potential role of m6A regulators in obesity and imply that expression of genes encoding writers, erasers and readers in adipose tissue may exert depot-specific effects on important pathways potentially related to adipogenesis or adipose tissue expansion. Genetic variation in m6A regulators adds an additional layer of variability to the functional consequences.

## Data Availability Statement

The original contributions presented in the study are included in the article/**Supplementary Material**. Further inquiries can be directed to the corresponding author.

## Ethics Statement

The ethics committee of the University of Leipzig and the Regional Committee for Medical and Health Research Ethics for South Eastern Norway have approved all study protocols and written informed consents were collected from all study participants. The patients/participants provided their written informed consent to participate in this study.

## Author Contributions

TR performed data analysis, statistical work and contributed to the manuscript draft. MD performed RT-qPCR analyses. TV, AC, and MK have critically contributed to the discussion. AT is the PI of the Sorbs cohort. MB is the PI of the adipose tissue cohort. YB initiated, conceived and designed the study, contributed to critical data discussion and wrote the final version of the manuscript. All authors contributed to the final manuscript by proof reading, editing and critical discussing the obtained results. All authors contributed to the article and approved the submitted version.

## Funding

TR and AC are funded by Helse-SørØst grants to YB. Further support of this work came from the Kompetenznetz Adipositas to MB (Competence network for Obesity) funded by the Federal Ministry of Education and Research (German Obesity Biomaterial Bank; FKZ 01GI1128). A grant by the Deutsche Forschungsgemeinschaft for a Collaborative Research Center (CRC 1052/2) further supported this work: ‘Obesity mechanisms’ project number 20993838 - SFB1052 (to AT and MB).

## Conflict of Interest

The authors declare that the research was conducted in the absence of any commercial or financial relationships that could be construed as a potential conflict of interest.

## Publisher’s Note

All claims expressed in this article are solely those of the authors and do not necessarily represent those of their affiliated organizations, or those of the publisher, the editors and the reviewers. Any product that may be evaluated in this article, or claim that may be made by its manufacturer, is not guaranteed or endorsed by the publisher.

## Supplementary Material

Supplementary Table 1 Primer sequences used for RT-qPCR analyses.

Supplementary Figure 1 Heatmap matrix of pairwise linkage disequilibrium. Heatmap matrix of pairwise linkage disequilibrium statistics (r2 and D´) of SNP markers in **(A)** the *YTHDF3* locus and **(B)** the *METTL3* locus. The figure was created by using “LDlink – An interactive webtool for exploring linkage disequilibrium in population groups (https://ldlink.nci.nih.gov/?tab=ldmatrix provided by NIH, National Cancer Institute). Data are represented for Europeans (CEU; Utah residents from North and Western Europe).

Supplementary Figure 2 Work flow and study design. Figure illustrates the stepwise study design.
