# Supplementary material for: m6A Regulators in Human Adipose Tissue - Depot-Specificity and Correlation With Obesity
Source: Front Endocrinol (Lausanne). 2021 Dec 7;12:778875. doi: 10.3389/fendo.2021.778875 (PMC8689137; doi:10.3389/fendo.2021.778875)
Supplement: Supplementary Table 1 — Primer sequences used for RT-qPCR analyses. [file Table_1.docx]

**SUPPLEMENTARY MATERIAL**

**Supplementary table 1.** Primer sequences used for RT-qPCR analyses.

| Target gene | Forward primer (5' -> 3') | Reverse primer (5' -> 3´) |
| --- | --- | --- |
| *METTL3* | CAAGCTGCACTTCAGACGAA | GCTTGGCGTGTGGTCTTT |
| *METTL14* | AGAGAACAAAGGAACACTGCCT | AATGAAGTCCCCGTCTGTGC |
| *WTAP* | TGCGACTAGCAACCAAGGAA | GTTGATCGCTGGGTCTACCA |
| *VIRMA/KIAA1429* | TCCGAGTCATACCCCCAGGA | AACAGGGGCACTTGGTTTGC |
| *FTO* | TGCCGAGGAACGAGAGCG | GGGGGTCAGATAAGGGAGCC |
| *ALKBH5* | TGTGCTCAGTGGATATGCTGC | GACTTTGTTTCCAACCGGGG |
| *YTHDF1* | CATGAAGCATGTCGGCCACC | TGACTGTCCAGTAAGGTAGGGC |
| *YTHDF2* | GCCAGCTACAAGCACACCA | CCGTTGCTGCAGTCTGTGT |
| *YTHDC1* | ATCTTCCGTTCGTGCTGTCC | GGACCATACACCCTTCGCTT |
| *IGF2BP2* | AGAAGTCATCGTGCCTCGTG | TTTCCCTGATCTTGCGCTGT |
| *PPIA* | CCGTGTTCTTCGACATTGCC | TTGTCTGCAAACAGCTCAAAGG |
