# Supplementary figures and images for: m6A Regulators in Human Adipose Tissue - Depot-Specificity and Correlation With Obesity
Source: Front Endocrinol (Lausanne). 2021 Dec 7;12:778875. doi: 10.3389/fendo.2021.778875 (PMC8689137; doi:10.3389/fendo.2021.778875)

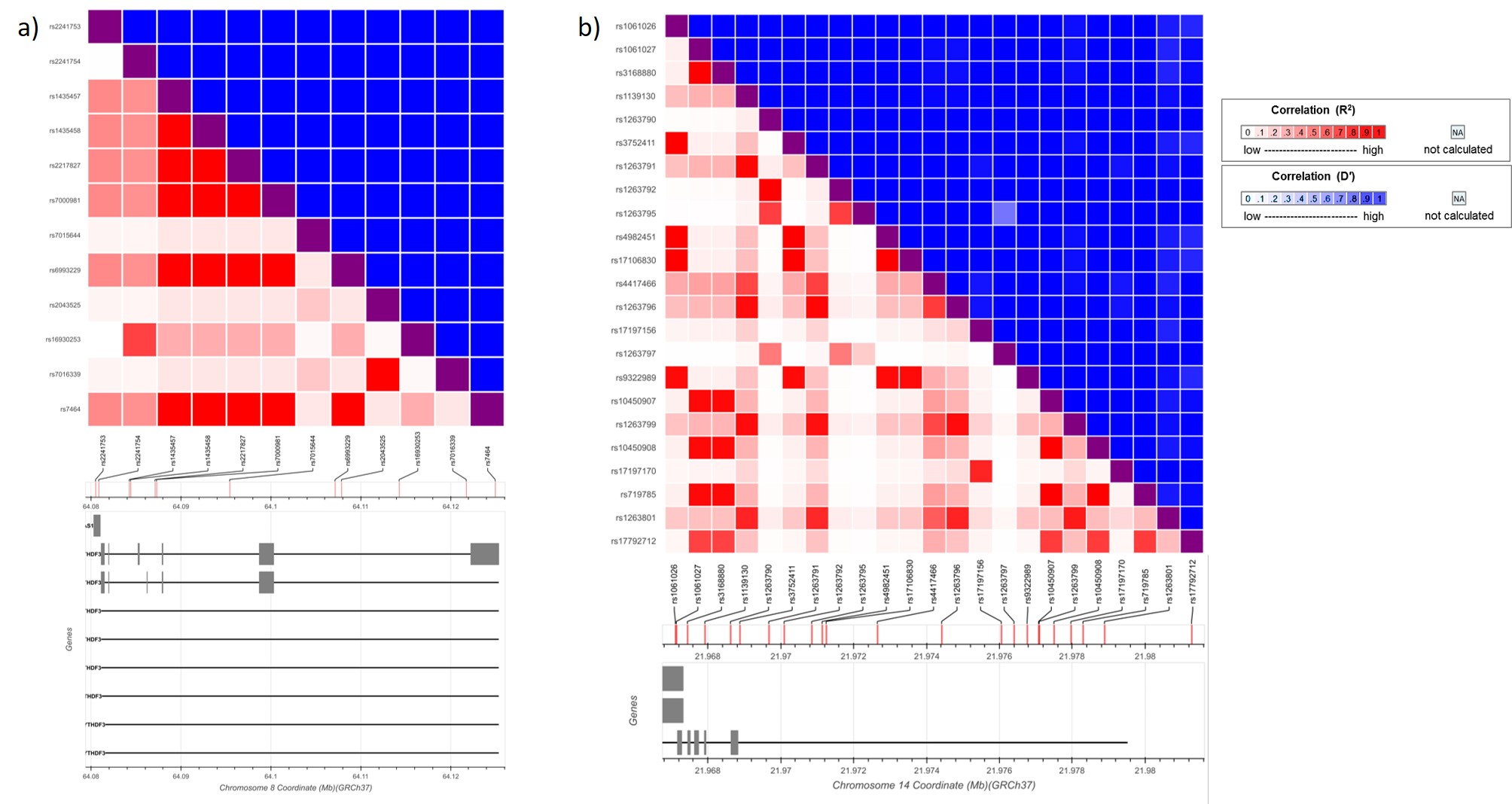

Supplement: Supplementary Figure 1 — Heatmap matrix of pairwise linkage disequilibrium. Heatmap matrix of pairwise linkage disequilibrium statistics (r2 and D´) of SNP markers in (A) the YTHDF3 locus and (B) the METTL3 locus. The figure was created by using “LDlink – An interactive webtool for exploring linkage disequilibrium in population groups (https://ldlink.nci.nih.gov/?tab=ldmatrix provided by NIH, National Cancer Institute). Data are represented for Europeans (CEU; Utah residents from North and Western Europe). [file Image_1.jpg]

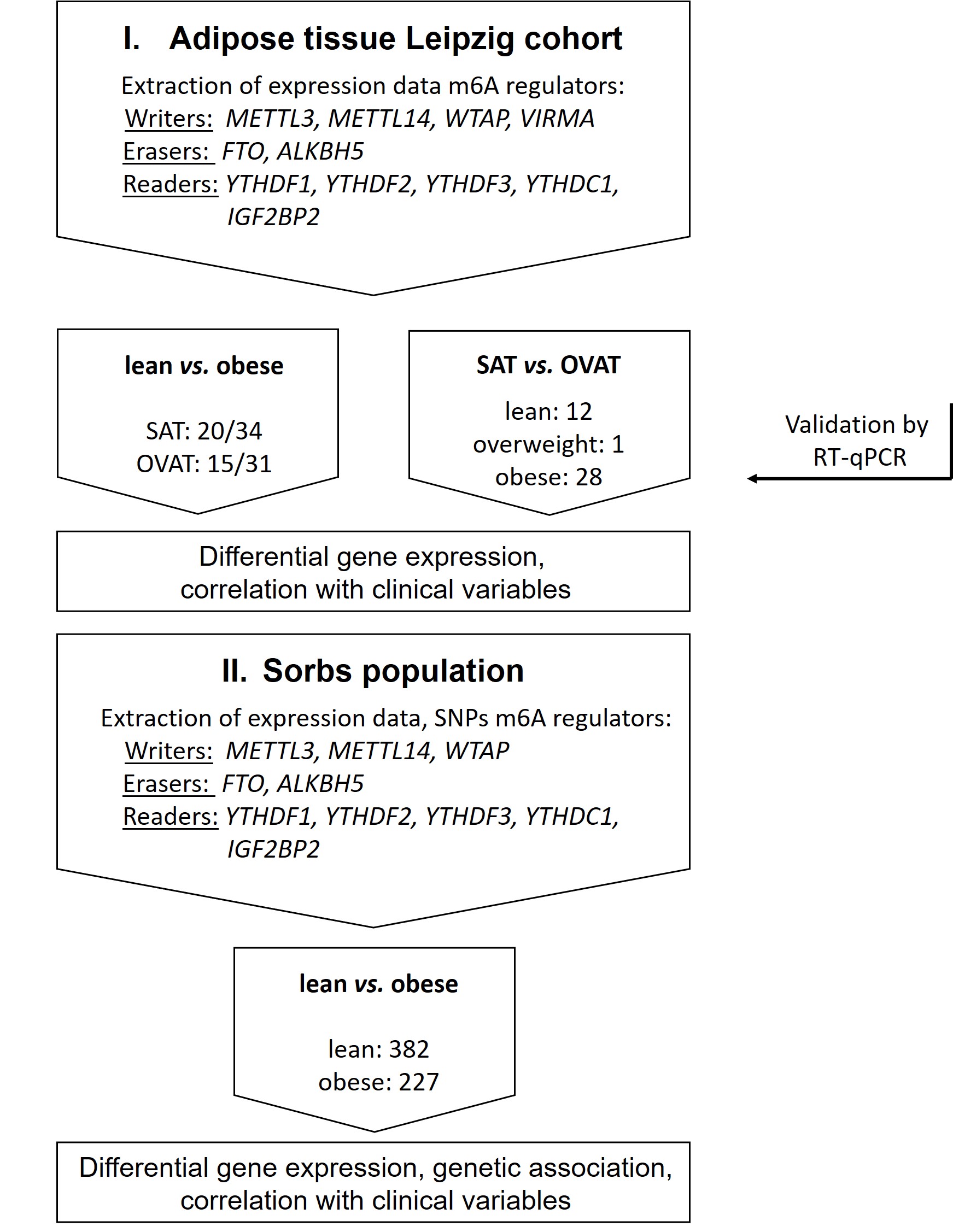

Supplement: Supplementary Figure 2 — Work flow and study design. Figure illustrates the stepwise study design. [file Image_2.jpg]
